# Accelerated Growth, Differentiation, and Ploidy with Reduced Proliferation of Right Ventricular Cardiomyocytes in Children with Congenital Heart Defect Tetralogy of Fallot

**DOI:** 10.3390/cells11010175

**Published:** 2022-01-05

**Authors:** Tatyana V. Sukhacheva, Roman A. Serov, Natalia V. Nizyaeva, Artem A. Burov, Stanislav V. Pavlovich, Yulia L. Podurovskaya, Maria V. Samsonova, Andrey L. Chernyaev, Aleksandr I. Shchegolev, Alexei I. Kim, Leo A. Bockeria, Gennady T. Sukhikh

**Affiliations:** 1 A.N. Bakulev National Medical Research Center of Cardiovascular Surgery, The Ministry of Health of Russian Federation, 121552 Moscow, Russia; seroroman@yandex.ru (R.A.S.); aikim@bakulev.ru (A.I.K.); leoan@bakulev.ru (L.A.B.); 2 National Medical Research Center for Obstetrics, Gynecology and Perinatology Named after Academician V.I. Kulakov of the Ministry of Healthcare of the Russian Federation, 117997 Moscow, Russia; niziaeva@gmail.com (N.V.N.); a_burov@oparina4.ru (A.A.B.); s_pavlovich@oparina4.ru (S.V.P.); y_podurovskaya@oparina4.ru (Y.L.P.); ashegolev@oparina4.ru (A.I.S.); sukhikh21@oparina4.ru (G.T.S.); 3 Department of Obstetrics, Gynecology, and Reproductology, Faculty for Postgraduate and Advanced Training of Physicians, First Moscow State Medical University Named after I.M. Sechenov, 119991 Moscow, Russia; 4 Research Institute of Pulmonology, Federal Medical-Biological Agency of Russian Federation, 115682 Moscow, Russia; samary@mail.ru (M.V.S.); cheral12@gmail.com (A.L.C.)

**Keywords:** tetralogy of Fallot, children, cardiomyocytes, differentiation, ploidy, Cx43, gap junction, proliferation, ultrastructure, interstitial tissue

## Abstract

The myocardium of children with tetralogy of Fallot (TF) undergoes hemodynamic overload and hypoxemia immediately after birth. Comparative analysis of changes in the ploidy and morphology of the right ventricular cardiomyocytes in children with TF in the first years of life demonstrated their significant increase compared with the control group. In children with TF, there was a predominantly diffuse distribution of Connexin43-containing gap junctions over the cardiomyocytes sarcolemma, which redistributed into the intercalated discs as cardiomyocytes differentiation increased. The number of Ki67-positive cardiomyocytes varied greatly and amounted to 7.0–1025.5/10^6^ cardiomyocytes and also were decreased with increased myocytes differentiation. Ultrastructural signs of immaturity and proliferative activity of cardiomyocytes in children with TF were demonstrated. The proportion of interstitial tissue did not differ significantly from the control group. The myocardium of children with TF under six months of age was most sensitive to hypoxemia, it was manifested by a delay in the intercalated discs and myofibril assembly and the appearance of ultrastructural signs of dystrophic changes in the cardiomyocytes. Thus, the acceleration of ontogenetic growth and differentiation of the cardiomyocytes, but not the reactivation of their proliferation, was an adaptation of the immature myocardium of children with TF to hemodynamic overload and hypoxemia.

## 1. Introduction

Tetralogy of Fallot (TF) is a congenital heart defect (CHD), the main anatomical components of which are misplaced aorta, ventricular septal defect, narrowing of the right ventricular (RV) outflow tract, and RV myocardial hypertrophy. TF is a polygenic heart disease resulting from autosomal dominant heterozygous mutations associated with the deletion of a fragment of chromosome 22 (del 22q11.2), and leading to a partial loss of function of transcription factors TBX1 and TBX5, NKX2.5, GATA4.5, 6 [1,2,3,4,5,6,7,8,9,10,11,12,13], as well as mutations of genes regulating the intracellular signaling pathway of Notch [14,15]. Some patients with TF have trisomy on chromosome 21 (Down syndrome) [1].

The peculiarity of hemodynamics in TF is associated with a narrowing of the pathway of blood outflow from the RV to the pulmonary artery, which leads to a decrease in blood flow in the lungs, low blood oxygen saturation (hypoxemia), and the discharge of some of the venous blood into the systemic circulation. In patients with TF, pressure and volume RV overload combined with myocardial hypoxemia occurs immediately after birth. Compensatory remodeling of the RV myocardium in TF is aimed at maintaining adequate hemodynamics and increasing blood oxygenation and consists of RV hypertrophy, increased blood oxygen capacity (increased hemoglobin level in the blood), and pulmonary hypertension. It is known that the proliferative activity of RV CMCs in patients with TF is associated with SaO_2_ parameters [16]. On the other hand, in a number of experimental studies it has been shown that pressure and volume RV overload, induced in the first days of postnatal development, similar to that arising in TF, leads to the activation of the expression of proliferative markers in CMCs, as well as to increased angiogenesis and, later, myocardial fibrosis [17,18,19]. One of the unresolved questions is whether the activation of the CMCs proliferation in the immature myocardium of children with TF can be caused and by what factors.

The first days of postnatal development are most important for the development of the myocardium, since at this time the transition from hyperplastic (proliferative) to hypertrophic growth of cardiomyocytes (CMCs) takes place. During this period, the activity of several cyclins and cyclin-dependent kinases (CDK), their inhibitors, and transcription factors, both positively and negatively regulating the cell cycle, change [20,21,22,23,24,25,26,27,28]. Significant morphological changes occur in the myocardium, characterized by a decrease in the proliferative activity of CMCs, an increase in their ploidy, size, and differentiation [19,28,29,30,31,32,33,34,35,36,37,38,39,40,41,42,43,44,45,46,47,48,49,50,51]. CMCs go through the last cell cycle without completing cytokinesis, which leads to an increase in the number of binucleated terminally differentiated CMCs at the G_0_ [28,29,34,35,36,52,53,54].

In recent years, the study of CMCs ploidy is an area of active interest due to its importance for the regenerative potential of the myocardium [28,55,56,57,58,59]. In addition to the classical methods for assessing the ploidy of human CMCs, it is proposed to use the technique of introducing a non-radioactive, non-toxic, stable isotope 15N-thymidine, which is included in DNA during the S-phase, and later to investigate 15N-thymidine-labeled CMCs in fragments of the myocardium removed during surgical correction of TF [60]. The appearance in children with TF of a large number of multinucleated CMCs in the first six months after birth is discussed as a consequence of the disruption of CMCs cytokinesis due to a decrease in the expression of Ect2 of the Hippo-Yap signaling pathway [28,61]. The polyploid nuclei formation is also associated with decreased expression of the nuclear lamina gene Lamin B2 (Lmnb2), which is necessary for the destruction of the nuclear envelope during the transition from prometaphase to metaphase and the completion of karyokinesis [58]. It is believed that an increase in CMCs ploidy negatively affects their regeneration capacity, unlike adult Zebrafish, where 99% of CMCs are mononuclear [62]. A population of small mononuclear diploid CMCs resistant to oxidative stress and retaining proliferative activity is revealed in the mouse myocardium [55,56]; there is a possibility of entry into the cell cycle of postnatal binuclear CMCs [57]. Interestingly, Hesse M. et al. (2021) [59] do not reveal a difference in the functions and plasticity of mononuclear and multinucleated CMCs, and a comparative analysis of the transcriptome demonstrated their similarity since mononuclear CMCs in most cases are polyploid and in a similar way to multinucleated CMCs react to focal lesions of the heart. When analyzing the plasticity of CMC, it is important to take into account not the number of nuclei, but the total ploidy of CMCs.

Myocardial hypertrophy of patients with CHDs develops in parallel with the growth of the body, all internal organs, and the heart, including [36,40,41,42,43,63,64,65,66,67,68,69,70,71,72]. At the same time, the morphological study of the myocardium of children with TF during the first years of life revealed an extremely heterogeneous morphological picture, in which the entire phenotypic continuum of ploidy and morphology variants of CMCs corresponding to the stage of their differentiation was observed. Yekelchyk M et al. (2019) [73] suggested that the main reason for the differences in CMCs morphology in a hemodynamically overloaded hypertrophied heart is heterogeneous vascular growth. But these data have been obtained in an experimental study of the myocardium of an adult mouse, and what happens in the myocardium of children with TF is not completely clear. The following questions remain unresolved: what is the reason for the differences in the CMCs morphology in children with TF (i); is the high ploidy of the CMCs any advantage for the adaptation of the children myocardium to hypoxemia and hemodynamic overload (ii); is it possible to significantly dedifferentiate the CMCs of children in the first years of life with TF with the return of the properties of the embryonic myocardium (iii)?

The present study is devoted to a comprehensive assessment of the morphological parameters of the myocardium in children with TF in the first years of life, with an emphasis on the ratio of changes in CMCs ploidy, their size, proliferative activity, ultrastructural and immunohistochemical signs of CMC differentiation with clinical data and age-related changes in the myocardium. We hypothesize that the main mechanism of adaptation of an immature myocardium to increased hemodynamic overload and hypoxemia is its accelerated maturation, which turns into hypertrophy upon reaching the limit of ontogenetic growth. We assume that the differentiation of CMCs in children with TF during the first years of postnatal development takes place in stages, therefore special attention is paid to the comparative analysis of the correlations of morphological signs and clinical parameters characterizing hypoxemia and hemodynamic overload of the RV outflow tract myocardium in children with TF younger and older than six months.

## 2. Methods

### 2.1. Patients and Samples

All potentially eligible patients with TF who receive care at the A.N. Bakulev National Medical Research Center of Cardiovascular Surgery (Moscow, Russia) were investigated. Intraoperative biopsies of the RV outflow tract myocardium removed during the correction of the defect of 69 patients with TF (including 31 patients aged three to six months and 38 patients aged 7–33.5 months, total 24 boys and 45 girls) were studied. The clinical characteristics of patients with TF are presented in a Table 1. As a control, we examined the material of autopsies of 12 age-matched (1–36 months, eight boys, four girls) patients without cardiovascular pathology.

### 2.2. Ethics Approval

The study was performed according to the World Medical Association Declaration of Helsinki and approved by the Ethical Committee of A.N. Bakulev National Medical Research Center of Cardiovascular Surgery (record №1 29.04.2021). All participating subject families provided written, informed consent before any study-specific evaluations of the patients’ biological materials were performed.

For histological and immunohistochemical studies, the material was fixed in buffered 10% formalin and embedded in paraffin.

### 2.3. CMCs Ploidy

To determine the ploidy of CMCs, a suspension of myocardial cells obtained by alkaline dissociation of paraffin-embedded tissue was used. Paraffin sections 60 µm thick were placed in special cassettes, dewaxed, and washed with distilled water. Then the samples were transferred into centrifuge tubes, incubated for 1.5 h at 37 °C with 50% KOH solution, centrifuged for 5 min at 2000 rpm, the supernatant was removed, the pellet was resuspended in distilled water and centrifuged for another 5 min at 2000 rpm. The last procedure was repeated two more times, after which a small amount of water was added to the precipitate. Tissue fragments were mechanically disaggregated, the cell suspension was spread over glass slides and dried. The nuclei of isolated cells were stained with DNA-specific fluorochrome DAPI (4′-6-diamidino-2phenylindole-HCl). The use of DAPI was based on its ability to bind to phosphate groups of DNA in a stoichiometric ratio, which makes it possible to calculate the relative DNA content [74,75]. Cell nuclei were examined using a Keyence BZ-9000 fluorescence microscope (Japan); the nuclei belonging to the CMCs were established by the cross striation of myofibrils. Fibroblasts and lymphocytes found in the same preparations were considered the standard of the diploid set of chromosomes. In each sample, 70–100 CMCs were examined, and the proportion of multinuclear myocytes was calculated. The CMCs’ ploidy (c) was calculated by comparing the fluorescence of the CMCs nuclei with those of the diploid nuclei of the reference cells. In multinucleated CMCs, the total ploidy of all nuclei was calculated.

### 2.4. Light Microscopy

On paraffin sections stained with hematoxylin and eosin, oriented longitudinally and passing through the CMCs nucleus, the transverse diameter of the CMCs and their nuclei were measured in at least 50 cells in each patient (×1000) using the Image-Pro Plus morphometric program (version 6.0.0.260; Media Cybernetics, Rockville, MD, USA). The measurement results were presented as the mean with standard deviation (mean ± SD). On the same sections on a four-point scale, the myofibrils assembly in the CMCs was determined: the sarcoplasm was filled with myofibrils (0 points), the zones of sarcoplasm with an incomplete assembly of myofibrils were less than 10% (1 point), 10–50% (2 points), more than 50% of the CMC area (3 points).

To identify the volumetric density of interstitial tissue in the myocardium, paraffin sections were stained by the Masson-Trichrome (Bio-Optica, Milano, Italy), the proportion of interstitial tissue (×100) was determined in 20 randomly selected fields of vision using the Image-Pro software.

### 2.5. Immunohistochemistry

The marker of gap junctions (GJs) protein connexin43 and CD34, a marker of hematopoietic stem cells and progenitor cells, expressed in endothelial progenitor cells, as well as in mature endotheliocytes, were identified immunohistochemically. Paraffin sections of the myocardium were dewaxed in alcohols, treated with hydrogen peroxide (37 °C, 15 min), washed with PBS (3 changes), then incubated with trypsin (37 °C, 30 min), washed with PBS (3 changes), and incubated with primary antibodies for connexin 43 rabbit polyclonal antibody (1:4000, C6219, Merck KGaA, Darmstadt, Germany) and CD34 mouse monoclonal antibody (RTU, QBEnd/10; Spring bioscience, Pleasanton, CA, USA). The antigen was detected using the ENVISION KIT HRP Rb/Mo DAB detection system (DAKO K 5007, Santa Clara, CA, USA) according to the manufacturer’s recommendations.

On longitudinal sections passing through the nucleus and intercalated disks of the CMCs, the length of the Connexin43 (Cx43)-positive zones of the sarcolemma on the lateral surfaces of the CMCs were determined. In the same CMCs (not less than 50 cells), the diameter and length were measured. The relative length of Cx43-containing GJs was expressed as a percentage of the doubled length of CMC. The density of CD34-positive endothelial cells was determined (in percent) in at least 25 fields of view (×400) on the TF myocardium preparations.

The slides were examined by light microscope Leica DMRB with Leica DFC495 camera and Leica PL FLUOTAR 10×/0.3 and Leica N PLAN 40×/0.65 (Leica Microsystems Gmbh, Buffalo Grove, IL, USA).

### 2.6. Transmission Electron Microscopy (TEM)

Fragments of the myocardium were fixed in a 2.5% glutaraldehyde and 1% paraformaldehyde in 0.1 M phosphate buffer (pH 7.4), postfixed in a 1.5% OsO4, dehydrated, and embedded in Araldite. Ultrathin sections (50–70 nm) were cut using ultramicrotome Leica ultracut UCT (Leica Microsystems Gmbh, Austria), collected on copper grids, contrasted with uranyl acetate and lead citrate, and examined using a Philips CM100 transmission electron microscope (Philips/FEI Corporation, Eindhoven, The Netherlands). The severity of the ultrastructural changes in the CMCs was assessed semi-quantitatively on a three-point scale: no changes (0), changes were detected in single cells (1), in half or more cells on a section (2).

### 2.7. Double Immunofluorescence

To determine proliferative active CMCs, paraffin sections were incubated with a mixture (1:1) of primary antibodies for Ki67 (1:200, rabbit anti-human, NCL-L-Ki67-MM1, Leica Biosystems, Buffalo Grove, IL, USA) and sarcomeric α-actin (Sarc α-act; 1:50, mouse anti-human, Abcam, Cambridge, UK), and then with a mixture (1:1) of secondary antibodies labeled with fluorochromes Alexa 488 (1:200, chicken anti-rabbit, Thermo-Fisher Scientific, Waltham, MA, USA) and Alexa Fluor 546 (1:200, donkey anti-mouse, Thermo-Fisher Scientific, USA). The nuclei were counterstained with 4′,6-diamidino-2-phenylindole (DAPI, Merck KGaA, Darmstadt, Germany). The preparations were examined using a confocal laser microscope Leica TCS SPE (Leica Microsystems Gmbh, Austria). The amount of Ki67^+^/Sarc α-act^+^ CMCs was counted. The area of the section was measured and the density of the CMCs was determined. The proportion of Ki-67^+^ CMCs/10^6^ CMCs in the myocardium was determined (median, min–max).

### 2.8. Statistical Analysis

Statistical analysis was performed using the Statistica 10.0 software program package. The corresponding data obtained through morphological research of myocardium in patients with or without TF were compared between the two groups using a non-parametric Mann—Whitney test, differences between groups were considered significant at a level *p* < 0.05. The relationship of morphological data with clinical and echocardiographic characteristics of patients was determined using the non-parametric Spearman correlation coefficient at *p* < 0.05 (r and *p* values were given in the text).

## 3. Results

### 3.1. CMCs Ploidy

**Figure 1:**
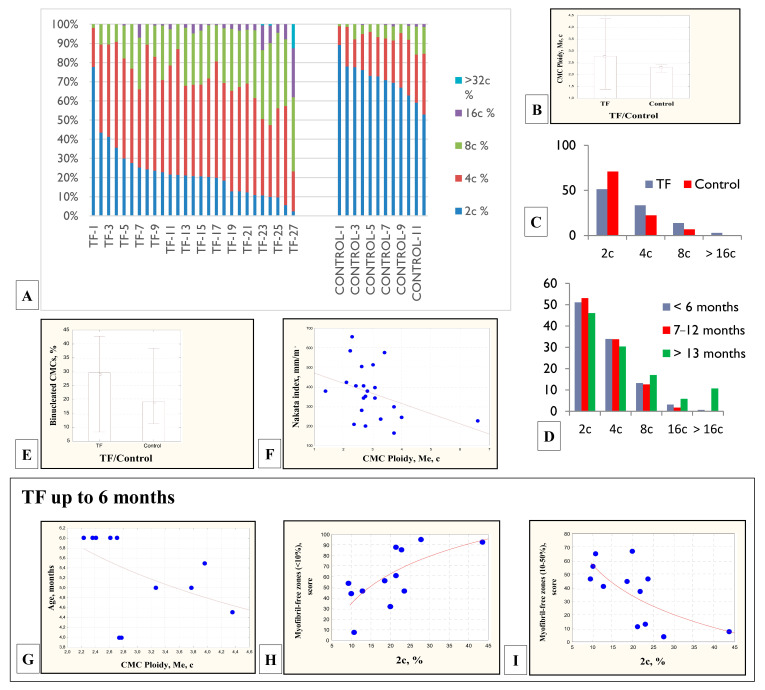
Distribution of CMCs of different ploidy classes in patients with TF and the control group. Comparison of ploidy (2c:4c:8c:16c:32c) of patients with TF and the control group (**A**). The ploidy of the CMCs in children with TF was significantly higher than the CMCs in the control groups (median, Mann-Whitney test, *p* < 0.05) (**B**). Comparison of CMCs ploidy classes in patients with TF and controls (**C**). Change in the ratio of different ploidy classes in patients with TF in different age groups: up to six months, 7–12 months, more than 13 months (**D**). Comparison of the proportion of binucleated CMCs in the group of children with TF and the control group (median, Mann-Whitney test, *p* < 0.05) (**E**). Inverse correlation of CMCs ploidy in children with TF with Nakata index (r = −0.44; *p* = 0.034) (**F**). In the myocardium of children under six months of age with TF: CMCs ploidy correlated inversely with age (r = −0.73; *p* = 0.007) (**G**). The myocardium of patients with a high percentage of diploid CMCs differentiated faster—the number of CMCs filled with myofibrils increased in it (r = 0.68; *p* = 0.015) (**H**) and the number of CMCs not filled with myofibrils decreased (r = −0.68; *p* = 0.014) (**I**).

The ploidy of RV CMCs in patients with TF increased significantly relative to the data of the control group (1.5–8.5c, median 3.3c vs. 2.3–3.6c, median 2.7c, Mann-Whitney test, *p* < 0,05) (Figure 1A,B). The distribution of CMCs by ploidy classes 2c:4c:8c:16c:32c in children with TF was 22:49:24:4.5:0.5, in the control group—71:22:6.2:0.8:0. The proportion of 2c CMCs in the myocardium of children with TF was reduced, while the proportion of 4c and 8c CMCs exceeded the data of the control group (Figure 1C). As patients with TF grew older, a gradual redistribution of the proportions of CMCs with different ploidy was noted. In patients under 6 months, 7–12 months, and older than 13 months, there was a decrease in the proportion of 2c and 4c CMCs and an increase in the proportion of 8c CMCs and 16c and higher (Figure 1D), which indicated increasing polyploidization of CMCs. Multinucleated CMCs nuclei, similar in size and shape, were usually located in the center at a short distance from each other. In general, multinucleated CMCs were characterized by a larger diameter than mononucleated ones (r = 0.71; *p* = 0.009), their number reached 42.8% of CMCs and exceeded the data of the control group (8.3–42.8% vs. 11.3–38.5%, Mann-Whitney test, *p* < 0,05) (Figure 1E). The CMCs ploidy of the children with TF was inversely correlated with the Nakata index (r = −0.44; *p* = 0.033), which characterizes the reduced hemodynamically significant pulmonary artery throughput (Figure 1F). A decrease in the index indicates hypoplasia of the pulmonary arteries, RV volume and pressure overload.

When examining the myocardium of children with TF under the age of six months, phenomena that were absent in older children were found. In this group, CMCs ploidy was inversely correlated with age (r = −0.73; *p* = 0.007) (Figure 1G), the myocardium with a high percentage of diploid CMCs differentiated more intensively—the number of CMCs filled with myofibrils increased (r = 0.68; *p* = 0.015) (Figure 1H) and the number of CMCs not filled with myofibrils decreased (r = −0.68; *p* = 0.014) (Figure 1I).

### 3.2. Morphometric Assessment of the CMCs Size

**Figure 2:**
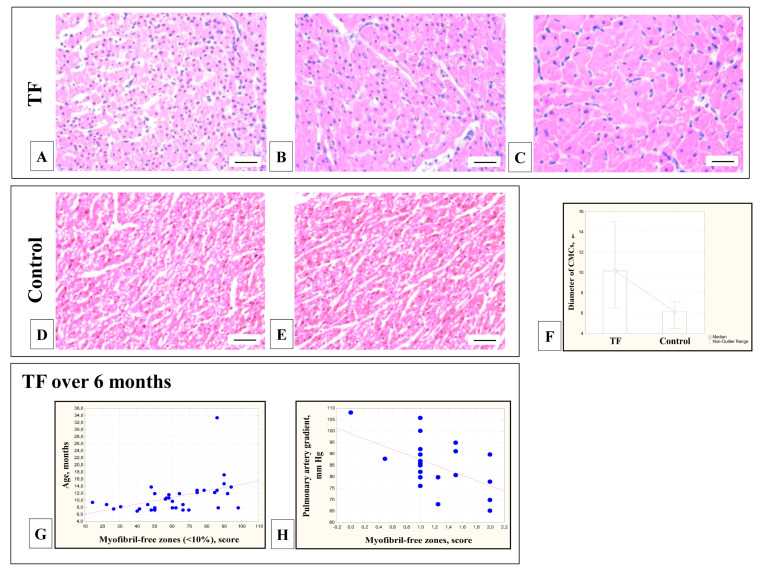
RV myocardium of patients with TF and in the control group. In the myocardium of patients with TF: significant variability of the CMCs diameter was revealed: on average, from 7.5 ± 2.4 μm (patient with TF, 10 months) (**A**) and 10.0 ± 1.5 μm (patient with TF, 5 months) (**B**) up to 10.8 ± 1.5 μm (patient with TF, 8 months) (**C**). In the control group: the diameter was 4.7–7.7 microns (**D**,**E**). Hematoxylin and eosin (×400) bar 20 µm. The diameter of the CMCs of children with TF significantly exceeded Table 1. (**F**). In children with TF over 6 months of age: The proportion of CMCs filled with myofibrils increased with age (r = 0.49; *p* = 0.003) (**G**), a high-pressure gradient on the pulmonary artery provoked accelerated differentiation with a decrease in the number of CMCs with sarcoplasm not filled with myofibrils (r = −0.39; *p* = 0.04) (**H**).

In the myocardium of children with TF, a significant increase in the size of the CMC was found in comparison with the control group (Figure 2A–E). The diameter of the CMCs in children with TF was 10.5 ± 2.1 μm, the diameter of the nuclei was 5.4 ± 0.8 μm, compared with the control group—5.7 ± 0.9 μm and 3.4 ± 0.5 μm (Mann-Whitney test, *p* = 0.0001) (Figure 2F). There was significant variability in the diameter of the CMCs and their nuclei in children with TF—from 6.5 to 16.7 μm and from 3.9 to 7.1 μm in different patients, while in the control group they varied only within 4.7–7.7 μm and 2.7–4.1 μm.

In children over 6 months of age with TF, an increase in the number of differentiated CMCs with sarcoplasm filled with myofibrils was age-related (r = 0.49; *p* = 0.003) (Figure 2G). A decrease in the number of undifferentiated CMCs, not filled with myofibrils, correlated with hemodynamic overload with a high-pressure gradient in the pulmonary artery (r = −0.39; *p* = 0.04) (Figure 2H).

### 3.3. Immunohistochemical Study of the GJ Protein Cx43

**Figure 3:**
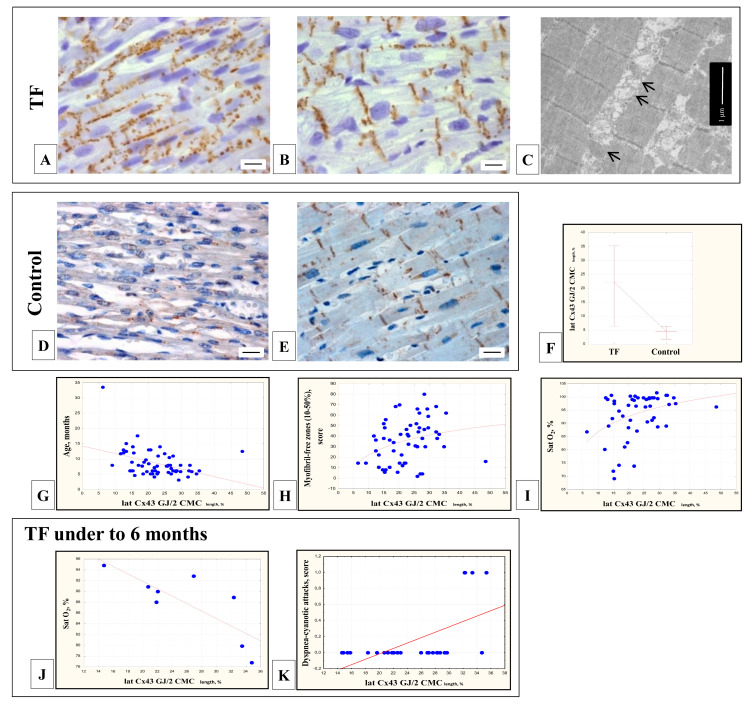
Connexin-43-containing (Cx43) gap junctions (GJs) in RV CMCs of patients with TF and the control group. In the myocardium of children with TF: Cx43-containing GJs were located both in the intercalated discs (**A**) and on the lateral sides, along the perimeter of the sarcolemma of the CMCs (**B**); a large number of binucleated CMCs. Immunohistochemical staining (×200), bar 10 µm. At the ultrastructural level, lateral GJs were found at the border of two CMCs in the form of concentric structures, following the bends of the CMCs sarcolemma (arrows) (**C**). In the control group: point Cx43-containing GJs were diffusely located along the perimeter of the sarcolemma (**D**). In the differentiated CMCs filled with myofibrils, the Cx43-containing GJs were localized predominantly in the intercalated discs (**E**). Immunohistochemical staining (×200), bar 10 µm. The relative length of lateral Cx43-containing GJs was significantly higher in the myocardium of patients with TF than in controls (Mann-Whitney test, *p* < 0.05) (**F**). The relative length of Cx43-containing GJs on the lateral surfaces of the CMCs decreased with age (r = −0.45; *p* = 0.0003) (**G**). The lateral arrangement of Cx43-containing GJs was characteristic of immature CMCs with incomplete differentiation, not filled with myofibrils (**H**). Lateral Cx43-containing GJs were less frequently observed in TF patients with low blood oxygen saturation, which suggested their accelerated differentiation (r = 0.34; *p* = 0.016) (**I**). In children with TF under six months of age, the lateral arrangement of Cx43-containing GJs was inversely correlated with blood oxygen saturation (r = −0.76; *p* = 0.028) (**J**) and positively correlated with a history of dyspnea-cyanotic attacks (r = 0.58; *p* = 0.002) (**K**).

In the RV myocardium of patients with TF of the first years of life, Cx43-containing GJs, as a rule, were located diffusely over the entire surface of the CMCs sarcolemma, which was typical for poorly differentiated CMCs, and in single CMCs of 13% of patients with TF—only in intercalated discs (Figure 3A,B). In some CMCs, Cx43-containing GJs were also exposed in the zones of additional intercalated discs connecting neighboring CMCs. At the ultrastructural level, we could also observe GJs connecting the lateral membranes of the adjacent CMCs (Figure 3C). In the control group, Cx43-containing GJs were located, as a rule, pointwise over the entire surface of the CMCs—both lateral surfaces and in the intercalated discs (Figure 3D,E). The relative length of Cx43-containing GJs on the lateral surfaces of the CMCs in the myocardium of the children with TF varied from 6.4 to 48.4% (median 22.3%) of the doubled CMC length and significantly exceeded this parameter in the control group (median 4.7%, 1.7–10.7%, Mann-Whitney test, *p* = 0.000001) (Figure 3F). It was necessary to note the more intense expression of Cx43 in the myocardium of the TF group as compared to the control group, as well as the heterogeneous distribution of Cx43-containing GJs in the CMCs of adjacent areas of the myocardium.

In the myocardium of children with TF (the entire group), the relative length of Cx43-containing GJs on the lateral surface of the CMCs correlated inversely with the age of patients (r = −0.45; *p* = 0.0003) (Figure 3G). The lateral position of Cx43-containing GJs was typical for CMCs with incomplete myofibrillogenesis (r = 0.36; *p* = 0.006) (Figure 3H). In patients with low blood oxygen saturation, the number of lateral Cx43-containing GJs was reduced, apparently due to their movement into the intercalated discs (r = 0.34; *p* = 0.016) (Figure 3I). It should be noted that in patients under the age of six months, in contrast to other children, the lateral location of Cx43-containing GJs, in contrast, was inversely correlated with arterial oxygen saturation (r = −0.76; *p* = 0.028) (Figure 3J) and positively with a history of dyspnea-cyanotic attacks (r = 0.58; *p* = 0.002) (Figure 3K).

### 3.4. Transmission Electron Microscopy (TEM)

**Figure 4:**
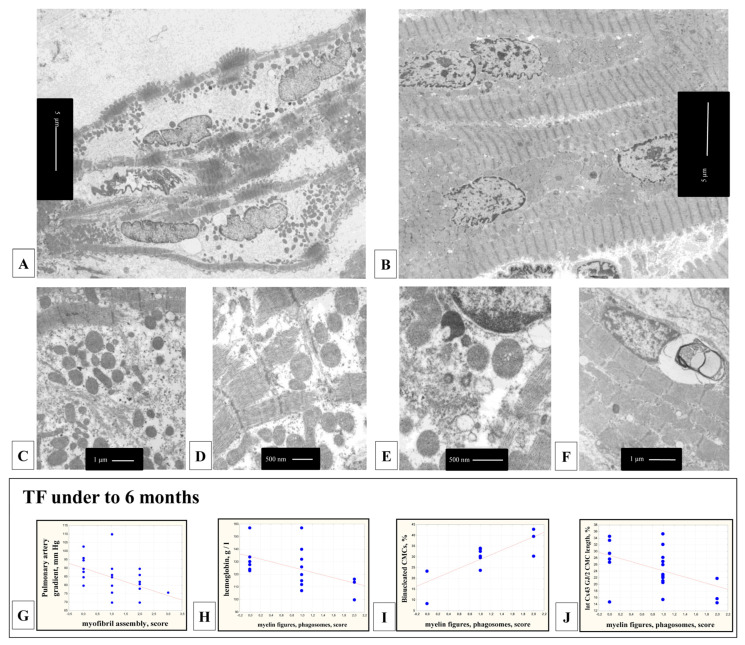
Ultrastructure of immature RV CMCs of children with TF. CMCs of an immature phenotype with electron-transparent sarcoplasm, not filled with myofibrils, with many small mitochondria and vacuoles in the sarcoplasm. Myofibrils were located in a thin layer along the periphery of the sarcoplasm, bar 5 µm (**A**). In the CMCs of a more differentiated phenotype, myofibrils filled most of the sarcoplasm, in the perinuclear zone there were mitochondria, glycogen granules, single lipofuscin granules, bar 5 µm (**B**). Assembly of myofibrils in the sarcoplasm of the CMC, bar 1 µm. (**C**). T-system channels at the level of Z-bands of organizing myofibrils, bar 0,5 µm (**D**). Paired centrioles, cisterns, and vesicles of the Golgi apparatus in the perinuclear zone of the CMC, bar 0.5 µm (**E**). Myelin figures, vacuole in the perinuclear zone of the CMC, bar 1 µm. (**F**). In children with TF under six months of age: the assembly of myofibrils was more common in the CMCs of patients with a low gradient on the pulmonary artery (r = −0.52; *p* = 0.01) (**G**); signs of dystrophic changes in ultrastructure (myelin figures, phagosomes) were found in the CMCs of children with low hemoglobin levels (r = −0.54; *p* = 0.025) (**H**), in the myocardium with a large number of multinucleated CMCs (r = 0.77; *p* = 0.006) (**I**)**,** in differentiated CMCs with a small relative length of lateral Cx43-containing GJs (r = −0.47; *p* = 0.32) (**J**).

In the CMCs of children with TF, there was the entire phenotypic range (continuum) of ultrastructural signs of various degrees of differentiation. In immature CMCs, the electron-transparent sarcoplasm was not filled with myofibrils, contained many small mitochondria, vacuoles, and only a thin layer of myofibrils was located under the sarcolemma (Figure 4A). In highly differentiated CMCs, myofibrils filled a large portion of the sarcoplasm In addition, there were large mitochondria, glycogen granules, and single lipofuscin granules (Figure 4B). In the sarcoplasm, foci of myofibril assembly were found, both in the center of the cell and at the periphery, under the sarcoplasm (Figure 4C). In undifferentiated CMCs at the level of the Z-bands of myofibrils, the channels of the T-system were identified (Figure 4D). Paired centrioles, cisterns, and vesicles of the Golgi apparatus were located in the perinuclear zone (Figure 4E). Signs of dystrophic changes in the CMCs ultrastructure (myelin-like membrane structures, phagosomes) in the myocardium of children with TF were rarely observed (Figure 4F). CMCs with myofibril-free zones were registered in 26 of 65 (40%) patients; their number decreased with age (r = −0.27; *p* = 0.025).

In children with TF under six months of age, the assembly of myofibrils in the CMCs was less frequently observed in patients with a high-pressure gradient in the pulmonary artery (r = −0.52; *p* = 0.009) (Figure 4G). Signs of dystrophic changes were detected in the CMCs of children with TF under six months of age with a low hemoglobin level (r = −0.54; *p* = 0.025) (Figure 4H), in the myocardium with a large proportion of multinucleated CMCs (r = 0.77; *p* = 0.006) (Figure 4I), as well as in differentiated CMCs with low relative length of lateral Cx43-containing GJs (r = −0.47; *p* = 0.32) (Figure 4J).

### 3.5. Morphometric Assessment of the Proportion of Interstitial Tissue

**Figure 5:**
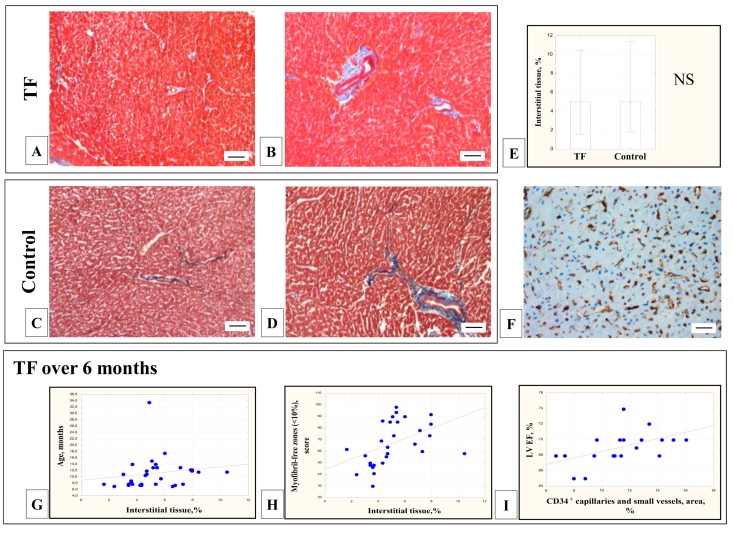
Interstitial tissue of the RV myocardium of children with TF and the control group. An insignificant proportion of interstitial tissue in the perivascular zone of the myocardium in children with TF (**A**,**B**) and the control group (**C**,**D**). Masson trichrome (×200), bar 10 µm. The proportion of interstitial tissue in the myocardium of children with TF and the control group did not differ significantly (**E**) (median, Mann-Whitney test, *p* > 0.05). CD34-positive capillaries and small vessels in the myocardial interstitium of a patient with TF. Immunohistochemical staining (×400), bar 20 µm (**F**). In children with TF over 6 months of age: the proportion of interstitial tissue correlated with age (r = 0.38; *p* = 0.03) (**G**) and increased with an increase in the number of differentiated CMCs, the sarcoplasm of which was filled with myofibrils (myofibril-free zones were 10%)(r = 0.64; *p* = 0.0001) (**H**). The density of CD34-positive small vessels and capillaries directly correlated with LVEF (r = 0.64; *p* = 0.003) (**I**).

In the myocardium of children with TF, an insignificant proportion of interstitial tissue was detected in the perivascular zones (Figure 5A–D), which did not differ significantly from the control group (5.5 ± 2.1% vs. 5.8 ± 3.2%, Mann-Whitney test, *p* > 0.05) (Figure 5E), did not correlate with the age of the patients and the CMCs’ diameter (*p* > 0.05). CD34-positive capillaries and small vessels created a network around the CMCs; their density varied in different patients from 6.6 to 17.2% and did not correlate with the clinical and morphological parameters of the patients.

In the myocardium of patients with TF older than six months, the proportion of interstitial tissue was increased with age (r = 0.38; *p* = 0.03) (Figure 5G) and in the myocardium of patients with a large proportion of differentiated CMCs whose sarcoplasm was filled with myofibrils (zones without myofibrils were up to 10%) (r = 0.64; *p* = 0.0001) (Figure 5H). Interestingly, in this group of patients, the density of CD34-positive small vessels directly correlated with LV (left ventricle) ejection fraction (EF) (r = 0.64; *p* = 0.003) (Figure 5I).

### 3.6. Immunofluorescent Detection of Proliferating CMCs

**Figure 6:**
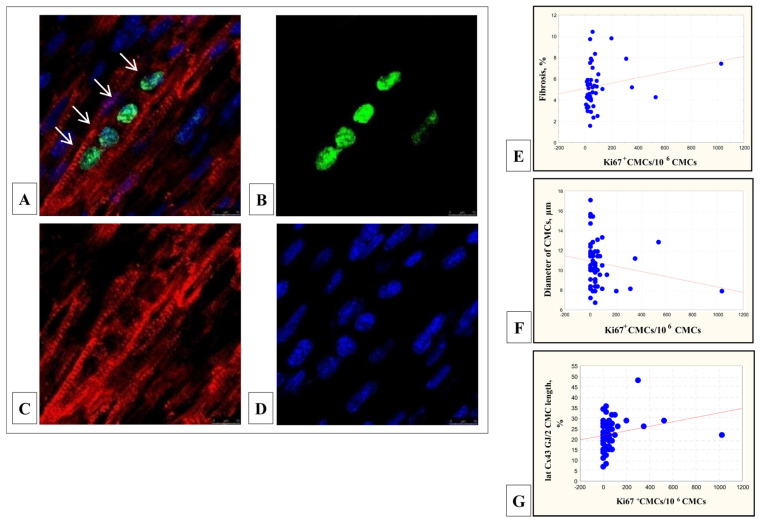
Proliferative-active Ki67^+^/Sarc α-actin^+^ CMC in the RV myocardium of patients with TF. In multinucleated CMC, all nuclei were Ki67-positive (arrows) (**A**). Alexa 488—anti-rabbit antibody to Ki67 (**B**); Alexa 546—anti-mouse antibody to Sarcomeric α-Actin (**C**); DAPI nuclei staining (**D**). Immunoconfocal microscopy, bar 10 мкм. Patient with TF, five months. Ki67-positive CMCs were detected in the myocardium with a large proportion of interstitial tissue (r = 0.33; *p* = 0.27) (**E**); in the myocardium with a smaller diameter of the CMCs (r = −0.31; *p* = 0.02) (**F**); in the myocardium with a greater relative length of lateral Cx43-containing GJs in the CMCs (r = 0.29; *p* = 0.02) (**G**).

In the myocardium of 43.0% (46/61) patients with TF, proliferative active CMCs (Sarc actin^+^) with Ki67-positive nuclei were identified. The average diameter of Ki67^+^/Sarc actin^+^ CMCs was 10.2 ± 3.2 μm, diameter of their nuclei—5.3 ± 1.5 μm, CMCs differentiation was consistent with the majority of CMCs in these patients (Figure 6A–D). The number of these cells varied significantly and ranged from 7.0 to 1025.5 (median 43) cells per million CMCs. In multinucleated CMCs, Ki67-positive staining is usually found in all nuclei. The number of Ki67^+^/Sarc actin^+^ CMCs correlated with the proportion of interstitial tissue (r = 0.33; *p* = 0.27), which indicated the development of the connective tissue frame in the children’s myocardium (Figure 6E). At the same time, the number of proliferatively active Ki67-positive CMCs decreased with an increase in the differentiation of CMCs: an increase in their diameter (r = −0.31; *p* = 0.02) (Figure 6F) and a decrease in the length of the Cx43-containing GJs on the lateral surfaces of the CMCs (r = 0.29; *p* = 0.02) (Figure 6G).

## 4. Discussion

The heart of children with TF works under hemodynamic overload and hypoxemia from birth. The patterns of reorganization of the RV myocardium in children with TF for the first time have been revealed in detail when studying the spectrum of morphological parameters presented below.

### 4.1. CMCs Ploidy

In this work, we have shown that in the RV myocardium of the children with TF, the ploidy of CMCs significantly exceeded the data of the control group because the proportion of 2c CMCs was reduced, and the proportion of 4c and 8c CMCs, on the contrary, exceeded the data of the control group. In addition, the individual CMCs ploidy in patients with TF varied to a greater extent than in the control group. The revealed inverse correlation between the increase in CMC ploidy and the Nakata index is of interest, since it illustrates the compensatory capabilities of the immature myocardium to increase its ploidy in response to hemodynamic overload.

During the neonatal period, according to the literature, the CMCs ploidy significantly differs in the myocardium of the human fetus, diploid CMCs predominate (average is 89.8 ± 4.3%), while tetraploid CMCs’—6.4 ± 1.9% [76]. In the period from 16 to 35 weeks of gestation, the number of DNA increases in proportion to the gestational age and the increase in fetal weight [77]. In children of the first years of life, 2c CMCs of the LV is about 94.3 ± 1.8%, tetraploid ones are 3.7 ± 2.2%; at the age of one to nine years, the proportion of 2c CMCs nuclei decreases to 85.4 ± 7.0%, and the number of 4c CMCs nuclei increases to 13.6 ± 7.1% [33,78]. With age, in line with the same trend, the proportion of diploid nuclei decreases to 61.9 ± 9.0%, tetraploid nuclei—increase to 31.8 ± 5.1% [76]. Thus, the ratio of CMCs of different ploidy classes changes—the number of 2c and 4c CMCs decrease, and the number of 8c CMCs and higher classes increases [76,78].

In a normal adult heart, the ploidy of left ventricular CMCs varies from 4c to 10c, averaging 6.2 ± 0.5c [79]. The total amount of DNA in the human myocardium, according to the cytophotometric study of the CMCs nuclei, increases with the increasing weight of the heart [78,80]. Thus, if in the first year of life, the proportion of polyploid left ventricular CMCs is 16.3 ± 5.2%, and at the age of 10–20 years—39.5 ± 6.9%, then in adults over 40 years of age, their proportion increases to 54.2 ± 5.8% CMCs [81]. According to other data, in an adult myocardium, the proportion of 2c CMCs is 18–46%, 4c CMCs—47–78%, 8c CMCs—4–23% of cells, 16c CMCs—about 1.1% [82].

In children with CHDs, myocardial hyperfunction stimulates an accelerated increase in CMCs ploidy: and if the number of 2c CMCs in the first year of life is 90–95% CMCs, with age their number decreases, while the proportion of 4c and 8c CMCs increases [32,33]. In the myocardium of children with CHDs at the age of 2–12 years, CMCs of all ploidy classes are presented—2c, 4c, 8c CMCs, and, in small numbers, 16c and 32c CMCs [63]. By the age of eight years, the amount of DNA and the number of diploid CMCs reaches the adult norm [32], the proportion of 4c CMCs increases to 19%, and 8c CMCs—up to 1% [33].

In addition, in the myocardium of children with TF and other CHDs during the first year of life, an increase in ploidy is manifested by an increase in multinucleated CMCs [28,29,36], the proportion of which can reach 42.0% [83] and 50–60% [28], similar to the one described by us. Mononuclear CMCs have a twofold higher ploidy compared to binuclear CMCs, most mononuclear CMCs are tetraploid [59]. Thus, a manifold increase in the amount of DNA and protein required to ensure the high contractile activity of myocytes is achieved. The impairment of karyokinesis and an increase in CMCs ploidy is believed to be due to a decrease in the level of expression of the nuclear envelope gene Lamin B2 (Lmnb2), which is necessary for the prometaphase—metaphase transition [58]. Disruption of CMCs cytokinesis in the period from 1 to 7 months after birth in patients with TF completes the CMCs cell cycle 3.3 times more often than division and leads to a decrease in the number of CMCs by 25% [28]. The main mechanism of CMCs cytokinesis disruption is repression of the gene ECT2, the activity of which is regulated by β-adrenergic receptors (β-AR) [28]. It has been shown that inactivation of β-AR genes and administration of β-blocker propranolol activates the division of CMCs in newborn mice; the same phenomenon is characterized by an increase in the amount of CMCs (endowment) and provides a compensatory effect after myocardial infarction in adults. Exposure to propranolol makes possible in vitro division of CMCs in TF patients [28]. Moreover, propranolol treatment from one month of age until surgical repair in children with TF has been proposed as a therapy to increase RV cardiomyocyte division and potentially reduce RV myocardial hypertrophy [84].

The inability of multinucleated CMCs to enter the cell cycle and divide is actively discussed in the literature. It is believed that in the myocardium of adult mice, the ability to proliferate is retained mainly in mononuclear diploid CMCs, their number varies in individuals from 2.3 to 17.0% [55,56]. Thus, the hemodynamically overloaded mouse myocardium contains 81% of mononuclear ethynyl-2-deoxyuridine (EdU)-labeled proliferating CMCs, 83.9% of which are diploid [56] (Patterson M. et al., 2017). It is assumed that the proportion of mononuclear 2c CMCs is inversely associated with the expression of the CMC-specific kinase Tnni3k [56,85] since in mice knocked out for the Tnni3k gene, the number of mononuclear 2c CMCs increases [56]. On the contrary, Hesse M. et al., (2021) [59] argues that in the peri-infarction zone of the myocardium, the behavior of mononuclear and binuclear CMCs is the same—they undergo hypertrophy with corresponding changes in gene expression, regardless of the number of nuclei or ploidy of the CMCs. Postnatal binuclear CMCs can re-enter the cell cycle and successfully undergo division by stimulating the pro-proliferative factors fetal bovine serum (FBS) or a combination of fibroblast growth factor 1 (FGF1) and p38 MAP kinase inhibitor (p38i) in only about half of the cases [57]. Moreover, the division of binuclear CMCs is accompanied by the formation of paired and unpaired centrioles and the formation of a multipolar and then pseudo-bipolar spindle of division, as a result, 72 ± 25% of daughter cells inherit an unequal number of centrioles [57].

Interestingly, according to our data, in patients with TF under six months of age, CMCs ploidy was inversely correlated with age, apparently due to delayed cytokinesis of CMCs, and accelerated differentiation was observed mainly in diploid CMCs. Apparently, in the first months after birth, the adaptation of the child’s myocardium to hypoxemia and hemodynamic overload was provided due to the contractile activity of diploid, rather than polyploid, CMCs. However, the analysis of the CMCs ploidy of all patients with TF (from 3,0 to 33,5 months) indicated significant polyploidization of the hemodynamically overloaded myocardium.

### 4.2. Morphometric Assessment of the Size of the CMCs

The diameters of the CMCs and their nuclei of the RV myocardium of children with TF significantly exceeded these parameters in the control group. In addition, it is necessary to note the focal location of changes and significant individual variability in the morphology of the myocardium of patients with TF, especially concerning the size of the CMCs and their nuclei. Probably, such differences are due to individual genetic backgrounds and the severity of the CHD. It is assumed that the mosaicity of morphological changes in the hemodynamically overloaded myocardium is expressed by differences in ploidy and hypertrophy of the CMCs. This may be due to local differences in oxygen content in the tissue and depends to a greater extent on the local tissue microenvironment and the supply of capillaries [73]. It has been previously shown that the expression of the hypoxia marker HIF1α in the LV myocardium in an experiment with aortic narrowing correlates inversely with the density of capillaries in the myocardium [73].

During ontogenetic development, from the first days of postnatal development, the RV CMCs increase in size. The dynamics of these changes have been described in the myocardium of children with TF aged from eight days to 16 years [42,43], from two to 23 years [64], and from four months to 13 years [36]. At the age of six days—eleven months, the size of the CMCs in children with TF still does not differ from the control group [40]; by the age of two years, the increase in the CMCs size is most pronounced [70], and by the age of three to six years, the growth of CMCs almost stops [37]. By the age of 4 years, the number of CMCs with a diameter of more than 15 μm is 58.7% [68], and the average CMCs diameter reaches 17.1 ± 2.1 μm [65]. In patients with TF, differentiated CMCs are considered hypertrophied when their diameter exceeds 20 μm [67] and their number significantly increases from four months to 13 years [36]. In adult patients with TF, the mean CMCs diameter is already 21.5–24.9 μm [72]. It should be noted that hypertrophy of the CMCs in patients with TF is a partially reversible process, and 2–36 years after surgery for radical correction of the defect, a decrease in the size of the CMCs was recorded compared to unoperated patients with TF, but these values exceed the CMCs size in control patients [65].

### 4.3. Immunohistochemical Detection of GJs Protein Cx43

In the RV myocardium of children with TF, Cx43-containing GJs were mainly found on the lateral surfaces and in the intercalated discs of the CMCs and, rarely, only in the intercalated discs. This lateral position of GJs was characteristic of the immature myocardium of patients with TF. It was confirmed by the inverse correlation of the relative length of lateral GJs with age and a positive correlation with incomplete myofibrillogenesis in the CMCs. The number of lateral Cx43-containing GJs in CMCs of children with TF significantly exceeded the data of the control group. A similar location was previously noted in the myocardium of children with TF from 3.5 weeks to 6 years [38,39]. Later, as the size and the degree of differentiation of the CMCs increased, mature intercalated discs with desmosomes, tight contacts, and GJs were formed. GJs predominantly redistributed from the lateral surfaces to the intercalated discs of CMCs. According to some data [86], this process in children with TF is completed by the age of six to seven years, according to others [87]—by the age of 12 years, and this is associated with the age of the patients and not with the severity of CHD.

In the present study, when analyzing the entire group of patients with TF (from 3.0 to 33.5 months), the redistribution of Cx43-containing GJs from the lateral surfaces to the intercalated discs correlated with the factor of hypoxemia—low blood oxygen saturation. However, in children with TF under the age of six months, we observed an inverse relationship—the lateral Cx43-containing GJs were more often detected in patients with low blood oxygen saturation and the presence of dyspnea-cyanotic attacks. Similarly, Kolcz J. et al. (2005) [38] noted a later movement of Cx43-containing contacts in the intercalated discs and a decrease in the expression of Cx43 in the myocardium of children with TF as compared to the control myocardium. The myocardium of children with TF under the age of 6 months is most sensitive to hypoxemia and responds with a delay in the differentiation of intercalary discs.

### 4.4. TEM Study of the CMCs

In the myocardium of children with TF, incompletely differentiated CMCs were identified, with sarcoplasm only partially filled with myofibrils, as described earlier [37,88]. There were foci of myofibril assembly, multiple small mitochondria, and centrioles in the cytoplasm. Correlation analysis revealed a decrease in the number of immature CMCs with age, indicating an increasing differentiation of the myocardium. Signs of dystrophic changes in the CMCs ultrastructure and autophagy (myelin-like membrane structures, phagosomes) in the myocardium of children with TF were rarely observed, although these substructures are described in CMCs in various forms of cardiovascular pathology [89,90,91,92,93,94,95,96,97,98,99]. They are considered as markers of insufficient CMCs adaptation to hemodynamic overload and hypoxemia.

During autophagy, damaged cell organelles are destroyed, surrounded by a double membrane, then fused with lysosomes and form autophagosomes, than damaged material are digested by lysosomal proteases with the release of amino acids for de novo protein synthesis. [96,100]. The membranes of autophagosomes include proteins Beclin-1 and Atg5 (autophagy-related protein 5), the hydrolytic protein of lysosomes cathepsin D is also secreted in autophagosomes; the structural and functional component of lysosomal membranes is LAMP-1 (lysosome-associated membrane protein-1) [94,100,101]. Autophagy is activated with age, which coincides with our data on a very insignificant number of autophagosomes and other dystrophic substructures in the myocardium of children with TF in the first years of life. Similar data have been obtained in the study of the myocardium of transgenic Atg5-deficient mice in normal conditions and under pressure overload. The impairment of autophagy does not cause any manifestations in Atg5-deficient hearts in early cardiogenesis, while in adult animals, cardiac hypertrophy, LV dilatation, and contractile dysfunction are developed [101].

Unexpectedly for us, the correlation analysis showed that in the myocardium of children with TF under six months of age, the number of myelin-like structures and phagosomes increased in differentiated and multinucleated CMCs in response to a decreased level of hemoglobin in the blood. Dystrophic changes, as we assume, can appear only in relatively mature CMCs. The myofibril assembly in the CMCs was also inhibited in response to the hemodynamic overload of the RV myocardium (with an increase in the pressure gradient on the pulmonary artery). Apparently, as in the case of the redistribution of Cx43-containing GJs, in the myocardium of children under 6 months of age, hypoxemia provoked a delay in CMCs differentiation.

### 4.5. Morphometric Assessment of Interstitial Tissue

An insignificant percentage of interstitial tissue was revealed both in the control RV myocardium and in children with TF in the first years of life—averaged about 5.5%. The data obtained coincided with those presented in the literature for children with TF aged one to 10 years, where fibrosis averaged about 3% and also did not differ significantly from its values in the control myocardium (3.3%) [40,42,66]. It has been shown that in children in the first years of life with CHDs as opposed to adult patients, there is no significant increase in fibrosis [40,67,68,70]. In our work, no relationship was found between the proportion of interstitial tissue in the myocardium of children with TF in the first years of life and the degree of CMCs hypertrophy, in contrast to the statements of other authors [36,43,64,72]. Moreover, we only found a relationship of the proportion of fibrous tissue with age and the assembly of myofibrils in the CMCs in the group of patients with TF over six months, when the myocardium was more differentiated. In fact, with age, in patients with TF, perivascular and interstitial fibrosis in the myocardium becomes one of the main morphological signs of pathological myocardial remodeling [36,43,64,67,68,70,72].

Low arterial oxygen saturation is another factor that might be expected to correlate with the development of fibrosis in the myocardium of children with TF. According to the literature, in one to two year-old patients with hypoxia and the cyanotic form of TF, the expression of the type Iα procollagen gene is increased fourfold, and the expression of the VEGF gene and glycolytic enzymes is two to five times reduced, compared with the non-cyanotic form of this CHD [69,71]. In 14-year-old adolescents with TF suffering from hypoxic attacks, a significant increase in RV myocardial fibrosis is also noted [64], while in adult patients with TF, the severity of myocardial sclerosis does not correlate either with age or with a pressure gradient in the RV outflow tract, or with hematocrit and arterial oxygen saturation [43,70]. According to our and published data, the sensitivity to hypoxia was characteristic of the immature myocardium of children with TF, but it manifested itself not in an increase in the proportion of fibrosis, but the redistribution of Cx43-containing GJs and some changes in the ultrastructure of the CMCs. The density of CD34-positive capillaries and small vessels in the myocardium of children with TF, as we found out, showed the variability, and only in the group of children with TF that were older than six months did we find a positive correlation with LV EF.. The development of the microvasculature in children with TF over six months of age reached the optimum density, which provided blood supply to the myocardium and its necessary contractile ability.

### 4.6. Immunofluorescent Detection of Proliferating CMCs

In the myocardium of children with TF, according to our data, proliferatively active Ki67^+^/Sarc actin^+^ CMCs were detected in less than half of the studied biopsies. Their number varied significantly in different patients—from seven to 1025 cells per million CMCs, and decreased with the growth and differentiation of the CMCs and the increase in the proportion of the interstitial tissue.

The transition from the neonatal to the postnatal period in the myocardium is accompanied by a decrease in the proliferative activity of CMCs with an increase in their differentiation. In the myocardium of children with CHDs, including TF, the number of proliferating CMCs decreases in the first months of postnatal development [41,50,51]. So, at the age of one to three months, Ki67^+^/troponin T^+^ CMCs are 0.55 ± 0.02%, at four to six months—0.05 ± 0.01%, and at seven–12 months—0.02 ± 0.005% [50]. According to other authors, in children with an interventricular septal defect, Ki67^+^/troponin T^+^ RV CMCs at the age of 0–six months is 0.23 ± 0.05%, at seven–12 months their number decreases to 0.02 ± 0.01%, and at the age of over 12 months, it is 0.01 ± 0.01% [51]. Similarly, in the RV myocardium of children with congenital malformations, the highest number of Ki67-positive CMCs is at the age of one month (15.0 ± 0.1% of cells), at the age from one month to two years their number decreases to 8.0 ± 0.3% of cells, and over age two—decreases to 5.0 ± 0.2% of cells [45]. There are rare observations on the proliferation of CMCs in the myocardium of children and adults in response to damage or overload of the heart. Single mitotically dividing CMCs have been found in the myocardium of a hypertrophied LV of a six-month-old child with uremia, and in 12- and 20-month-old children with myocardial hypertrophy caused by coarctation of the aorta [102]. Proliferating CMCs have been registered in the LV myocardium of adult patients with aortic valve stenosis, ischemic heart disease, hypertrophic and dilated cardiomyopathies [103,104,105,106,107].

In the myocardium of laboratory animals (mice and rats), the maximum proliferative activity of CMCs has been detected in the first week of postnatal development. It decreases later, but the ploidy of myocytes increases due to the formation of multinucleated CMCs [19,30,31,34,44,47,49]. The peak of DNA synthesis (up to 10% of labeled CMCs nuclei) and the maximum number of Ki67-positive CMCs nuclei are noted in the mouse myocardium on the fourth day of postnatal development, which later decreases to 0% [34,44,49]. In three to five day-old mice, DNA synthesis mainly occurs in mononuclear diploid CMCs, and after 1.5 days or more, in polyploid binuclear CMCs [49]. The number of proliferating CMCs decreases from 8.5% (four-day-old mice) to 5.5% (seven-day-old mice) and 1.4% (14-day-old mice). In the same period, the intensity of BrdU-label incorporation into the CMCs nuclei decreases from 9.6% (three-day-old mice) to 0.94% (seven-day-old mice) and 0.02% (14-day-old mice) mice); later, only single labeled nuclei are found [44]. This is followed by a phase of an increase in the number of nuclei, which reaches a maximum by the seventh day, and a phase of CMCs polyploidization, the peak of which falls on the 14th day [49]. Thus, during the first four days after birth, the number of mouse LV CMC increases by an average of 25–40%, in the period between 14 and 18 days—it increases by another 40% and does not change further [48,108].

Experimental studies show that myocardial injury in the first week of postnatal development stimulates the greatest proliferative activity of CMCs. Myocardial injury caused to one-day-old mice (myocardial infarction induced by ligation of the anterior descending coronary artery/resection of a part of the myocardium of the LV apex/injection of diphtheria toxin causing cardiac death) leads to regeneration of the damaged area seven–21 days after injury [109,110,111,112,113]. During this period, markers of CMCs proliferation are recorded in the perinfarction zone—Ki67, phosphorylated histone 3H, BrdU, an increase in the number of binuclear CMCs [110,113,114]. At the same time, a similar injury inflicted on seven to eight-day-old mouse hearts leads to scar formation after one to two weeks [110,111,112]. The statistical probability of the proliferative response of the mouse CMCs to myocardial injury gradually decreases between days two and 21 [48]. Similarly, the narrowing of the abdominal aorta in 2-day-old rat hearts causes activation of DNA synthesis on the fifth day, which by the 10th day leads to an increase in the size of the CMCs [115]. In rats with iron deficiency anemia at the age of less than one month, LV myocardial hypertrophy is accompanied by an increase of 30–35% in the total amount of DNA and activation of DNA synthesis in the CMCs in comparison with control animals [116], the total number of CMCs nuclei in the LV increases by 17%, and also the number of CMCs with two nuclei increases by 17% [117]. While in adult rats with anemia, myocardial hypertrophy is accompanied by an insignificant short-term increase in the DNA amount and the number of ^3^H-thymidine-labeled CMCs nuclei [116,118], while later it decreases and returns to the original values [119].

The search for histological and molecular markers of the transition from prenatal to postnatal development in mammals is an extremely interesting line of research. One of the possible explanations for the abrupt cessation of CMCs division during this period of ontogenesis is the theory of the existence of a limiting number of cell divisions [120]. The arrest of the CMCs cell cycle in the early postnatal period is believed to be associated with a significant decrease in telomerase expression. So, on the fifth day of postnatal development of the rat myocardium, telomerase activity is 20% of the prenatal level and on the 20th day, it drops to zero [121]. In 10–15-day-old mice, the dysfunction of telomerase with a corresponding decrease in the length of the CMC telomeres [122] coincided with the termination of their proliferative activity. The number of cells expressing telomerase is reduced 20 times in adult animals (0.012 ± 0.003%) compared with newborns (0.24 ± 0.08%) but increases 6.45 times after the cryoinjury of the heart in adult animals [123]. CMCs of telomerase-deficient mice with dysfunctional telomeres (G3 *Terc−/−*) are usually multinucleated and demonstrate a decrease in proliferative activity in response to cardiac cryoinjury [122]. On the contrary, increased expression of telomerase in the mouse myocardium leads to an extension of the CMCs cell cycle and further CMCs hypertrophy [124].

It is also assumed that the block of mitosis in postnatal CMCs and the transition to the postmitotic phase and polyploidization of CMCs in the first weeks after birth is due to a decrease in the expression of the protein regulating CMCs cytokinesis—Ect2 and a decrease in RhoA-GTP activation [28]. In the same period, reverse regulation of cyclins and CDKs associated with the transition between the G1/S and G2/M phases and the positive regulation of cyclins and CDKs associated with the G1 phase is identified [18,20,21,23,27]. Increased expression of CDK1 and cyclin B1, as well as CDK4 and cyclin D1, induces the proliferation of fetal CMCs [27]. Expression of cyclin G1, which is a transcriptional target for p53, coincides with the transition from proliferation to polyploidization and hypertrophy of CMCs. Cyclin G1 promotes DNA synthesis but inhibits cytokinesis in neonatal CMCs, which leads to an increase in the of multinucleated CMCs [23].

Other mechanisms are known to control the arrest of CMCs in the G_0_ phase of the cell cycle, such as the induction of CDKs inhibitors represented by members of the INK4 family (p15, p16, p18, p19 and CIP/KIP family (p21, p27, p53, and p57) [17,18,19,21,22,23,24]. It has also been shown that the Notch signaling pathway, due to its action on cyclin D, initiates the entry into the cycle of resting embryonic stem cells and neonatal ventricular CMCs [125]. The YAP effector of the Hippo pathway plays an important role in the activation of CMCs proliferation, active version of YAP is capable of reprogramming the adult mouse CMCs and transferring them to a more fetal and proliferative state [126]. In turn, the transcription factors E2F1, E2F2, E2F4, CASZ1, GATAT4, Tbx20, and FoxM1 are involved in the upregulation of cyclins. In contrast, the transcription factors Meis1, MEF2D, FoxO1, and FoxO3 inhibit cell cycle progression by increasing the expression of p21, p27, p15, and p16 proteins [25,26].

In recent years, the role of epigenetic factors in the regulation of gene expression during the transition from prenatal to postnatal heart development, in particular DNA methylation, has been actively studied. DNA methylation causes changes in the chromatin structure by covalent modification of DNA and histones, causes repression of fetal heart genes, and activates the expression of sarcomeric components of mature CMCs. In the perinatal period, methylation and expression of about 440 CMCs genes are known to change [127,128]. Thus, during this period, the fetal troponin I1 isoform (Tnni1) is repressed by methylation, and, on the contrary, the postnatal Troponin I3 isoform (Tnni3) is activated through demethylation [127,129]. In the myocardium of patients with TF, high levels of methylation of the NKX2-5 and HAND1 promoters [130] and low levels of methylation of the TBX20 promoter are found in comparison with the control group, which is regarded as one of the factors in the pathogenesis of this CHD [130,131,132].

The regulation of postnatal myocardial development also involves microRNAs—small untranslated RNAs, which consist of 18–25 nucleotides and serve as powerful regulators of gene expression by inhibiting translation or stimulating mRNA degradation [26,133]. Among the microRNAs that positively regulate the CMCs cell cycle are miR-31a, miR-499, miR-302-367, miR-590, miR-199a, miR-204. While miR-1, miR-15, miR-16, miR-26a, miR-29a, miR-30a, miR-34a, miR-133a, and miR-141 negatively regulate the cell cycle by altering the expression of cyclins D, E, A and B, and CDK1, 2 and 6 [26,134]. The microRNAs miR-208a, miR-208b, and miR-499 control the expression of myosin heavy chain isoforms in CMCs and provide synthesis switching from α (αMHC) to β (βMHC) isoform of myosin heavy chains after birth [135].

In children with CHDs, the myocardium undergoes significant hemodynamic overloads from birth and later in postnatal development, when the period of dynamic balance between hyperplasia and hypertrophy has already been completed. Apparently, during this period, no pathological factors can cause significant dedifferentiation of the CMCs with the return of the properties of the embryonic myocardium. This explains the small amount of proliferatively active Ki67^+^/Sarc actin^+^ CMCs in the myocardium of children with TF and their significant decrease as the size and the differentiation of myocytes increase. Although, when assessing our results, it should be taken into account that the proliferative activity of epy RV CMCs is usually lower than in other parts of the myocardium [136].Moreover, the myocardium of children with TF, in addition to hemodynamic overload, suffers from insufficient blood oxygen saturation. According to some authors, the size of the RV CMCs in children with TF in the first years of life can correlate with arterial oxygen saturation, hemoglobin level, hematocrit, and RV end-diastolic pressure [68,70], which was not found in this work. In addition, according to data of a number of authors, insufficient blood oxygen saturation can stimulate the proliferative activity of CMCs. Interesting patterns were revealed by the researchers Ye L, et al. [16], who showed that the CMCs of patients with TF with moderate SaO_2_ have the highest proliferative activity, compared with patients with higher or lower SaO_2_. At the same time, the results of experimental studies show that proliferatively active BrdU, H3Ser10, and aurora B kinase-labeled CMCs are registered in the heart of animals kept under hypoxic conditions, and the scar size after myocardial infarction induced by ligation of the left coronary artery is significantly less than in animals under conditions of normoxia [137]. Myocardial injury caused by clamping of the aorta or coronary artery with subsequent hypoxia in adult rats leads to an increase in the number of 3H-thymidine- and BrdU-labeled LV CMC [138,139,140].

For example, in animals kept under hypoxic conditions, the size of the scar after the left coronary artery ligation-induced myocardial infarction is significantly less than under normoxia and proliferatively active BrdU-, H3Ser10-, and aurora B kinase-labeled CMCs are recorded [136]. Myocardial injury caused by clamping of the aorta or coronary artery [137,138,139] with subsequent hypoxia in adult rats leads to an increase in the amount of ^3^H-thymidine- and BrdU-labeled atrial and ventricular CMCs [138,139].

Prolonged narrowing of the pulmonary artery stimulates an increase in the average volume of CMCs and the number of their nuclei by 41% [117]. The pressure overload of the mouse LV myocardium caused by aortic clamping also induces hypertrophy and proliferation of CMCs. (Moreover, the number of small BrdU-positive proliferative-active CMC in this case, according to various sources, increases by almost five–50 times compared to the control [136,141]. Similarly, experimentally induced myocardial anemia in sheep embryos leads to an increase in heart weight, an increase in the size and number of ventricular binuclear CMCs, and an increased proliferation (Ki67-pos) of CMCs, compared with control [142]. Obviously, the results presented by other authors confirm that hypoxemia promotes the activation of CMCs proliferation; however, in this work, such correlations were not revealed. At the same time, in the present work, a statistically significant tendency of high proliferative activity of the myocardium with small-diameter CMCs was found, just as in the mouse myocardium it is the mononuclear small CMCs that retain proliferative activity and are resistant to oxidative stress [55].

Based on the results of the studies presented above, one could assume that hypoxemia should stimulate the activation of CMCs proliferation in children with TF. However, this work did not show that the adaptation of the myocardium of children with CHD to hemodynamic overload and hypoxemia causes an increase in the proliferative activity of the CMCs. Nevertheless, we revealed a relationship between low blood oxygen saturation and hemodynamic overload of the myocardium with parameters characterizing an increase in the RV myocardium differentiation—an increase in CMCs ploidy, redistribution of Cx43-containing GJs on the lateral surfaces of the CMCs, and, finally, with changes in the interstitial tissue of the myocardium.

## 5. Conclusions

In the immature myocardium of children with TF in the first years of life, the acceleration of ontogenetic growth and differentiation of CMCs is the basis for their adaptation to hemodynamic overload and myocardial hypoxemia. A complex of morphological changes characteristic of the accelerated ontogenetic development of the CMCs in the first years of life was registered in the RV outflow tract myocardium. An increase in the ploidy and size of CMCs, active assembly of myofibrils, maturation of intercalated discs with the accumulation of Cx43-containing GJs in them, ultrastructural signs of incomplete differentiation of CMCs, and a decrease in the proliferative activity of CMCs were revealed. It should be noted that in the myocardium of children with TF under six months of age, the differentiation of CMCs had its features—the level of CMCs ploidy were decreased, probably due to CMCs cytokinesis, diploid CMCs were primarily filled with myofibrils, sensitivity to hypoxemia was manifested by delayed differentiation of intercalated discs and myofibril assembly and the appearance of ultrastructural signs of degenerative changes in the CMCs. An increase in the proportion of interstitial tissue did not play a significant role in myocardial remodeling in children with TF, but increased with differentiation of the myocardium in children over six months of age. Thus, in children with TF in the first years of life, the differentiation of the RV CMCs was determined not so much by their age as by hemodynamic indicators of the severity of the CHD and the severity of hypoxemia. The sequence of changes in the morphology of CMCs in children with TF did not change during differentiation; the changes concerned only the rate of ontogenetic growth.

## Figures and Tables

**Table 1:** Clinical parameters of the children with TF.

Clinical Parameters	M ± SD (Min–Max)
Age, months (min–max)	8.4 ± 4.4 (3.0–33.5)
Left ventricular ejection fraction (LVFE), %	68.7 ± 2.6 (62–79)
The end-diastolic volume of the left ventricle, mL	45.7 ± 8.8 (33–70)
The systolic pressure gradient between the RV and pulmonary artery, mm Hg	85.9 ± 9.8 (68–110)
Sat O_2_, %	91.9 ± 11.5 (40–101.7)
Hemoglobin, g/L	133.1 ± 16.1 (100–162)
Hematocrit, %	39.2 ± 4,4 (30–49.5)
Nakata index, mm/m^2^	375.2 ± 105.3 (152.6–696)

## Study Limitations

The limitations of the TEM study of the intact human heart were associated with the impossibility of fulfilling the requirement for tissue processing (rapid tissue fixation to avoid autolysis) in the autopsy material. Therefore, the control material was examined only at the light-optical level.

## Abbreviations

CMC	Cardiomyocyte
TF	tetralogy of Fallot
RV	Right ventricle
LV	Left ventricle
GJ	gap junction
Cx43	Connexin 43
CHD	Congenital heart defect
TEM	Transmission electron microscopy
